# Systematic review of metabolism and safety aspects of monosodium glutamate intake in infants and lactating mothers: a scientific and regulatory perspective

**DOI:** 10.3389/fnut.2026.1825764

**Published:** 2026-06-22

**Authors:** Trina Sengupta, Hruda Nanda Mallick, Raj Kumar Yadav

**Affiliations:** 1 Department of Physiology, ESI-PGIMSR Medical College and Hospital, Joka, Kolkata, India; 2 Retired, Gurugram, India; 3 Department of Physiology, All India Institute of Medical Sciences, New Delhi, India

**Keywords:** evidence-based practice, flavoring agents, food safety, human milk, infant, newborn, maternal exposure, sodium glutamate

## Abstract

**Background:**

Monosodium glutamate (MSG) is widely used as a flavor enhancer, and its safety has been extensively studied for decades, providing a robust evidence base. Scientific discourse has focused on its effects on neurodevelopment, metabolism, and specific populations, such as neonates, and these effects have been carefully evaluated. Regulatory agencies worldwide have periodically revisited the evidence base, emphasizing the need for updated, balanced assessments. Following PRISMA 2020 guidelines, we searched PubMed, Embase, Scopus, Web of Science, and the Cochrane Library up to August 2025. Eligible studies included randomized controlled trials (RCTs), observational studies, preclinical investigations, and regulatory assessments. Two independent reviewers screened 3,800 records, with moderate inter-rater agreement (Cohen’s kappa = 0.40), resolving all discrepancies through consensus. Data were synthesized narratively due to heterogeneity. Risk of bias was assessed using standard tools (RoB 2, Newcastle–Ottawa, SYRCLE, and narrative appraisal).

**Results:**

Thirty-five studies and regulatory reports met inclusion criteria: 2 RCTs, observational studies (*n* = 16), preclinical studies (*n* = 8), and regulatory evaluations (*n* = 9). An additional 39 references provide an informed background and mechanistic context. Human studies have consistently shown efficient MSG metabolism, stable glutamate levels in breast milk, irrespective of maternal intake, and no adverse outcomes at dietary exposures. Regulatory evaluations confirmed safety, and animal studies confirmed safety at dietary levels, with effects observed only under extreme, non-physiological conditions. The risk of bias was low to moderate in human studies but higher in animal studies due to the indirectness of exposure models. Regulatory evaluations were primarily of low risk.

**Conclusion:**

Current evidence indicates that MSG is safe. Future research may further refine our understanding of developmental physiology and sensitive populations, building on the strong foundation of current evidence. A balanced interpretation of evidence is crucial for guiding public health communication and regulatory decision-making.

**Systematic review registration:**

https://www.crd.york.ac.uk/PROSPERO/view/CRD420251239310.

## 1 Introduction

Human milk is essential for infant nutrition, providing a diverse range of bioactive substances that shape growth, immune function, and long-term metabolic health. Within its non-protein nitrogen fraction, free amino acids (FAAs) constitute a small proportion by weight, but they have substantial physiological effects. Glutamate and glutamine dominate this fraction, together accounting for up to 70% of total FAAs as measured across different ethnicities by analytical methods (1). Their concentrations increase throughout the lactation period. Free glutamate rises from approximately 1.25 mM in preterm milk to around 1.75 mM by 3–6 months postpartum, and glutamine increases by more than 300% during the same period. This pattern, compared with the trajectories of other FAAs, points to a regulated secretory mechanism aligned with a human infant’s developmental needs (1–3).

Glutamate is a multifunctional amino acid. In the central nervous system (CNS), it serves as an excitatory neurotransmitter and metabolic intermediate, while glutamine supports intestinal barrier integrity, lymphocyte proliferation, and nitrogen balance (4, 5). Clinical studies have shown that glutamate may also function as a gut-brain satiety signal. Infants consuming glutamate-enriched formula displayed lower intake volumes and longer inter-meal intervals while maintaining healthy growth (6). These findings offer a physiological explanation for the well-documented ability of breast-fed infants to regulate their energy intake. This behavior may support healthy long-term weight regulation (4, 6).

From a regulatory perspective, the sodium salt of L-glutamic acid, commonly known as MSG, has been extensively studied and is widely recognized as a safe and effective flavor enhancer in food. Global bodies such as the Joint FAO/WHO Expert Committee on Food Additives (JECFA), the European Food Safety Authority (EFSA), Food Standards Australia and New Zealand (FSANZ), and the US FDA have repeatedly concluded, based on methodologically rigorous reviews, that MSG is safe for the general population (5, 7–9). The European Food Safety Authority (EFSA) Panel on Food Additives and Nutrient Sources Added to Food conducted a comprehensive re-evaluation of glutamic acid and its salts as food additives (5). The Panel established an acceptable daily intake (ADI) of 30 mg/kg body weight per day for this group, based on a no-observed-adverse-effect level (NOAEL) of 3,200 mg monosodium glutamate (MSG)/kg body weight per day and applying the default uncertainty factor of 100. The NOAEL was derived from a preliminary behavioral neurodevelopmental study conducted in 1979 (10). However, shortly after the EFSA opinion was published, the author re-examined the relevance of his own findings and concluded that there was no evidence that dietary MSG causes developmental neurotoxicity, and therefore no justification for altering the safety status of dietary MSG (11). In addition, the results of the Vorhees et al. (1979) study used by EFSA to establish the NOAEL were not adopted by other regulatory authorities in their safety assessments of glutamates (12). In 2019, EFSA issued a call for technical data to obtain more detailed information on the actual uses and use levels of glutamic acid and its salts, with the aim of improving the estimation and characterization of exposure to glutamates from all sources (13). More recently, the European Commission requested a new scientific opinion from EFSA on the safety of glutamic acid and its salts as food additives (14). Although there is no global requirement for labeling, the Food Safety and Standards Authority of India (FSSAI) has opted for precautionary labeling for pregnant women and infants (15).

Infants and lactating mothers are often considered a sensitive population in food safety assessments due to ongoing physiological maturation, distinct exposure patterns, and the presence of critical developmental windows. In early life, immature organ systems and age-dependent differences in absorption, distribution, metabolism, and excretion (ADME) may alter internal exposure, while rapid development of the brain, immune, and endocrine systems may increase susceptibility to adverse effects during specific periods. Infants also have relatively high dietary intake per body weight and limited dietary sources, primarily breast milk or formula, further shaping exposure conditions (16). However, such sensitivity does not necessarily translate into increased risk for all substances and must be evaluated on a case-by-case basis. For MSG, the JECFA re-evaluated in 1987 and assigned an acceptable daily intake (ADI) of “not specified,” removing earlier restrictions for infants. JECFA concluded that, although neonatal rodents may show greater sensitivity to glutamate-induced neurotoxicity, evidence from primate studies, long-term and reproductive toxicity studies, as well as human physiological data, including limited placental transfer and efficient metabolism of dietary glutamate (e.g., from human milk), did not indicate an increased health risk for infants (17). Thus, the maternal–infant dyad is a distinct biological unit that cannot be accurately represented by general adult population data and requires a comprehensive, systematic evaluation that considers its metabolic and developmental stages. This underscores the importance of integrating mechanistic, toxicokinetic, and species-relevant evidence when evaluating sensitive populations, and reinforces the need for systematic, case-by-case assessment rather than reliance on generalized assumptions of vulnerability.

## 2 Methods

### 2.1 Study design

#### 2.1.1 Eligibility criteria

Studies were included if they satisfied the following parameters:

- Population (P): Infants, neonates, pregnant women, lactating mothers, validated preclinical developmental models.
- Exposure (I): Glutamate occurring naturally or via supplementation.
- Comparators (C): Placebo, non-supplemented formula, or control group equivalents.
- Outcomes (O): Indicators of metabolism, toxicity, placental transfer, neurodevelopment, feeding behavior, and regulatory evaluation.
- Study Design (T): Randomized controlled trials (RCTs), cohort or case–control studies, systematic reviews, toxicological investigations, and official regulatory assessments.

Editorials, letters without data, and narrative commentaries were excluded.

### 2.2 Systematic review protocol

This systematic review was conducted according to the Preferred Reporting Items for Systematic Reviews and Meta-Analyses (PRISMA 2020) guidelines to ensure methodological transparency and reproducibility (18). The primary objective was to assess the metabolism, safety, and regulatory implications of MSG exposure in neonates, infants, pregnant women, and lactating mothers. Given the heterogeneity of the available evidence, encompassing randomized, observational, preclinical, and regulatory data, a narrative synthesis was employed, as outlined by the Synthesis Without Meta-analysis (SWiM) framework (19).

The present study is a systematic review of previously published literature and publicly available regulatory reports. As this research did not involve the collection of primary data from human participants or the use of animal subjects by the authors, institutional ethical approval and informed consent were not required. The review was conducted and reported in strict accordance with the Preferred Reporting Items for Systematic Reviews and Meta-Analyses (PRISMA 2020) guidelines to ensure methodological transparency and integrity.

### 2.3 Search strategy

A comprehensive literature search across multiple electronic databases was conducted to identify relevant studies. The databases searched included PubMed/MEDLINE, Embase, Scopus, Web of Science, and the Cochrane Library. Additional manual searches were conducted to include grey literature and other relevant sources. The initial search yielded 3,800 records, comprising 1,200 from PubMed/MEDLINE, 900 from Embase, 600 from Scopus, 500 from Web of Science, 400 from the Cochrane Library, and 200 from other sources. The search spanned all years from 1968 to August 2025 and employed both MeSH headings and free-text terms, using the following Boolean combinations:

“monosodium glutamate” OR “MSG” OR “glutamic acid” OR “glutamate salts.”

AND “infant” OR “neonate” OR “newborn” OR “pregnancy” OR “lactation” OR “breast milk.”

AND “safety” OR “toxicity” OR “blood–brain barrier” OR “placental transfer” OR “development.”

Reference lists of eligible reviews, authoritative government reports, and agency assessments (e.g., JECFA, EFSA, FSANZ, FSSAI, and US FDA) were scrutinized to identify additional sources. Duplicate records were removed using Zotero 6.0.

### 2.4 Study selection

**Figure 1:**
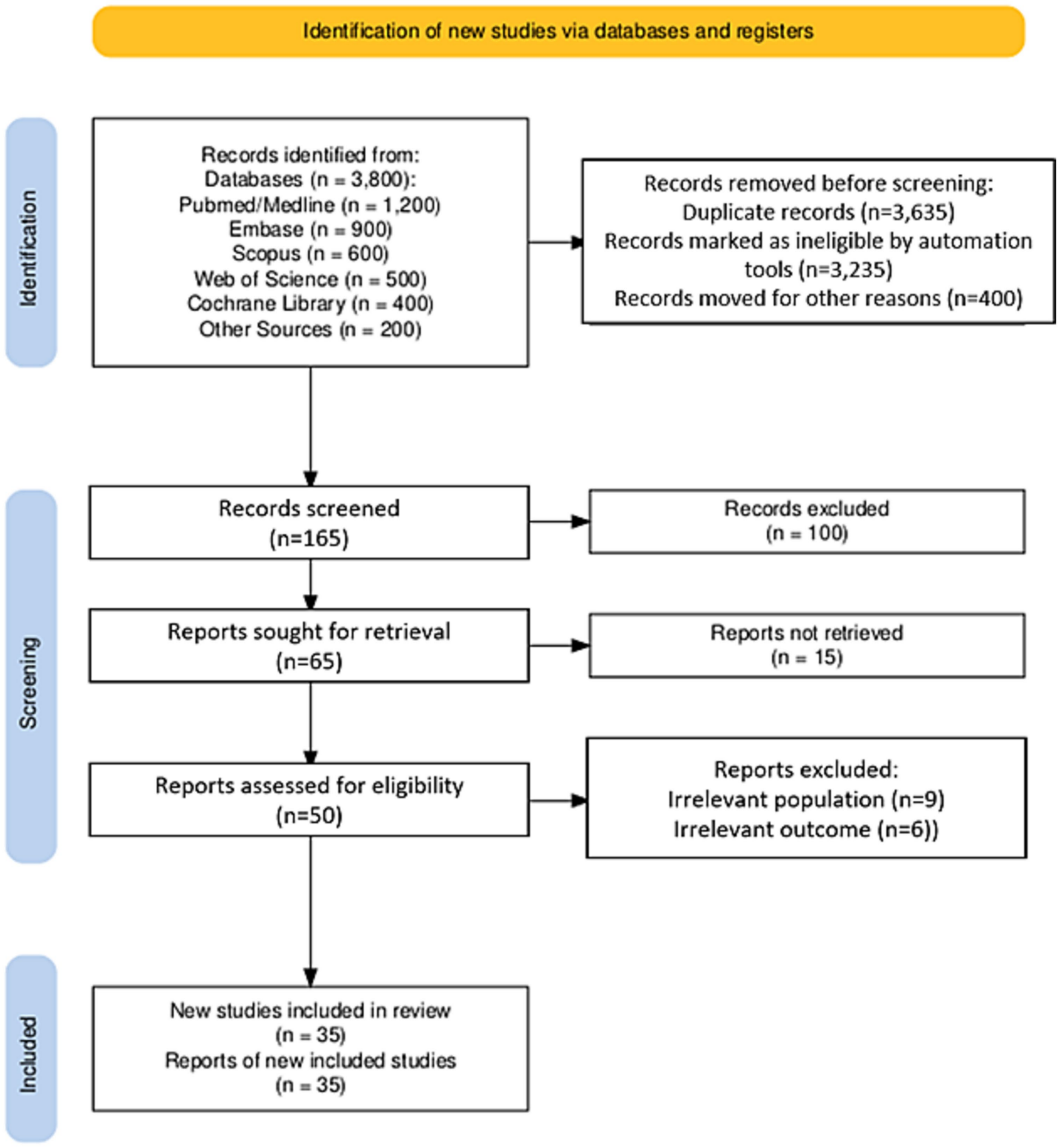
PRISMA 2020 flow diagram of study selection. A total of 3,800 records were identified from electronic databases. Following duplicates removal by automation tools (*n* = 3,650), and manually (*n* = 400), leaving (*n* = 165) for screening. Fifty full-text articles were sought for retrieval, of which 35 were assessed for eligibility. Studies were excluded primarily due to irrelevant populations or outcomes. Thirty-five studies met the inclusion criteria and were included in the final review.

Two independent reviewers (HNM and TSG) screened all records by title and abstract; full texts were retrieved for studies meeting the inclusion criteria. Discrepancies were resolved through consensus with a third reviewer. Inter-rater agreement for title and abstract screening was moderate (kappa = 0.40), with all conflicts resolved by consensus. The screening outcomes were depicted in a PRISMA 2020 flow diagram (Figure 1). After removing duplicates using automated tools (*n* = 3,235) and manually excluding records (*n* = 400), 165 records remained for screening (18). Screening was performed based on titles and abstracts, resulting in the exclusion of 100 records deemed irrelevant. Full-text reports were sought for the remaining 50 studies; however, 15 reports were not retrieved. Thirty-five full-text articles were then assessed for eligibility against the inclusion criteria and included in the qualitative synthesis.

### 2.5 Data extraction and synthesis

Structured data extraction templates were used to capture the following fields: publication year, population description, sample size, intervention/exposure dose, outcome measures, and principal findings. Given substantial heterogeneity and non-comparability across experimental designs, quantitative meta-analysis was not feasible; thus, a narrative synthesis approach was employed following SWiM guidelines. When appropriate, outcomes were organized into five domains:

i) glutamate content in human milk and infant intake,
ii) taste perception and feeding behavior,
iii) glutamate metabolism and handling,
iv) placental transfer and fetal exposure,
v) safety and regulatory evaluations.

### 2.6 Risk of bias assessment

The methodological quality of the studies was assessed by study type. RCTs were analyzed using the Cochrane risk of bias tool version 2 (RoB 2). Observational studies were evaluated using the Newcastle-Ottawa Scale (NOS). The SYRCLE risk-of-bias tool was used for animal studies. The regulatory and expert panel reports were appraised narratively for methodological rigor, transparency, and comprehensiveness.

The two included RCTs were judged to have a low-to-moderate overall risk of bias. Random sequence generation and outcome reporting were adequately described; however, challenges with blinding and small sample sizes raised concerns, particularly regarding outcome measurement and deviations from the intended interventions. The 16 observational studies were rated as moderate RoB, primarily due to limitations in exposure assessment and the potential for residual confounding. Outcome reporting was generally consistent, while methodological aspects, such as dietary MSG quantification and co-variable adjustment, were identified as areas for further improvement.

**Figure 2:**
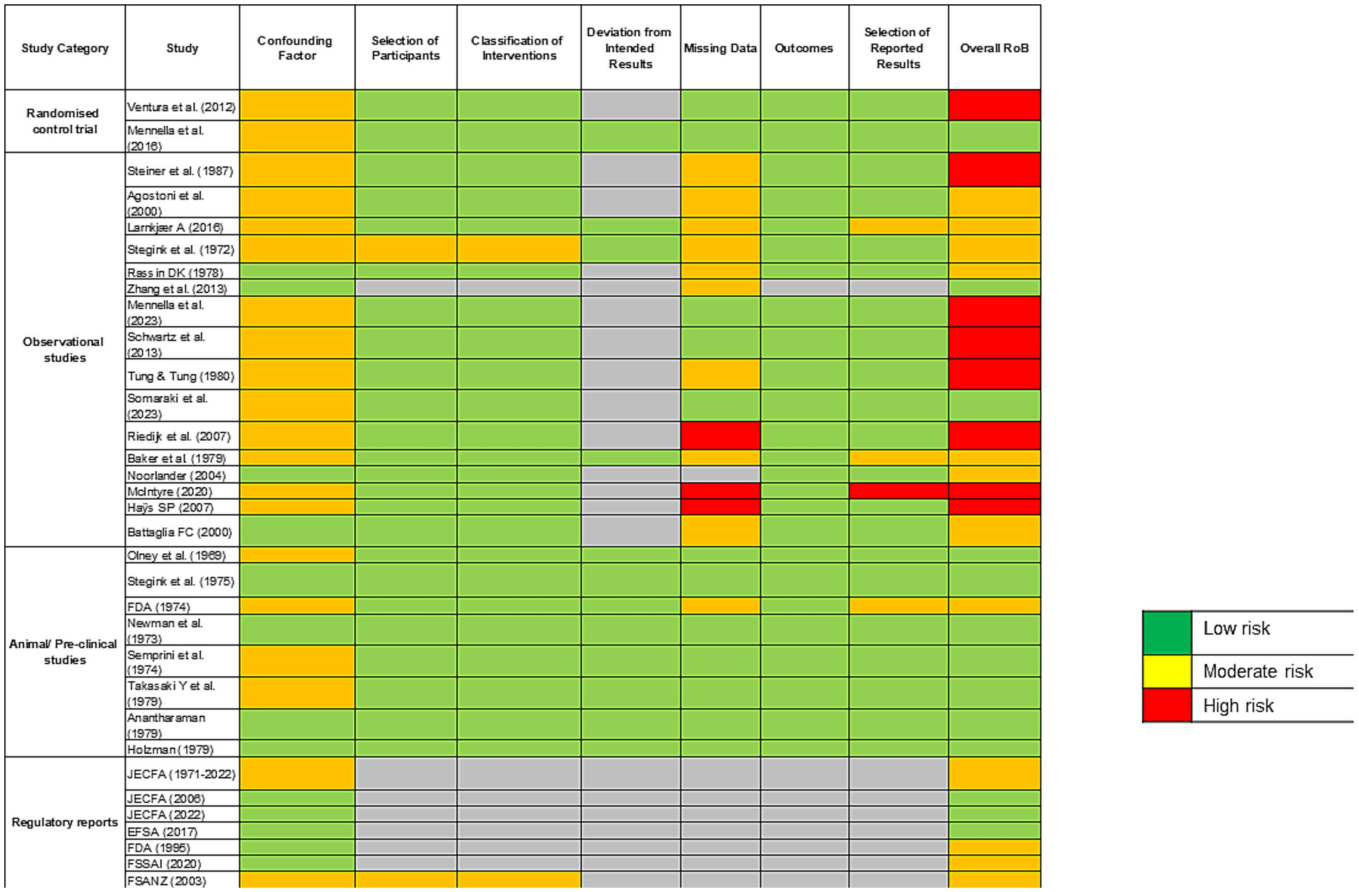
Risk of bias assessment across included studies and regulatory reports. The figure summarizes the risk of bias (RoB) evaluation for all sources included in the review, grouped into four categories: randomized controlled trials, observational studies, preclinical/animal studies, and regulatory reports. Individual studies are listed along the *y*-axis, and domains of bias are displayed across the *x*-axis. Each cell is color-coded to indicate the level of risk: green = low risk, yellow = moderate risk, and red = high risk. Assessment domains include confounding, participant selection, intervention/exposure classification, deviations from intended interventions, missing data, outcome measurement, and selection of reported results. The overall RoB for each study is presented in the final column. This figure provides a comparative visualization of methodological quality across evidence streams included in the review.

The eight preclinical/animal studies consistently noted methodological limitations. Several older studies provided limited detail on allocation or blinding procedures, as many experiments used supra-physiological doses or non-physiological routes of administration. These methodological features mean the findings are not directly generalizable to human dietary exposure. Finally, nine regulatory and expert evaluations (e.g., JECFA, EFSA, FDA, FSSAI) were appraised separately. Global agencies such as JECFA and EFSA demonstrated high methodological transparency and were judged to be at low risk of bias. The FSSAI’s precautionary stance on MSG in infant foods reflected context-specific caution rather than methodological issues. Overall, the RoB assessment (Figure 2) indicated that human clinical and observational evidence were at low to moderate risk of bias, animal studies were at higher risk due to indirectness, and regulatory evaluations were generally at low risk, with some national variation in interpretation.

## 3 Results

### 3.1 Study selection and characteristics

The systematic search identified 3,800 records, of which 35 studies met eligibility criteria after screening, duplicate removal, and full-text assessment (PRISMA flow diagram, Figure 1). These studies comprised two randomized controlled trials (RCTs), 16 observational studies, 8 preclinical animal studies, and 9 regulatory and expert evaluations. In addition to these 35 included studies, 31 further references were cited throughout the manuscript (Table 1). These papers provided background on the physiological mechanisms of glutamate metabolism, general trends in breastfeeding and infant feeding practices, perceptions of umami, and methodological or regulatory considerations. Although not included in the formal evidence pool synthesized in the results, these elements were considered essential to ensure a comprehensive and contextually informed review.

**Table 1:** Characteristics and key findings of included studies.

S No.	Study type	Author (s)	Year (s)	Population/model	Sample size	Route of administration	Dose (mg/kg bw/day or total)	Duration of exposure	Intervention/exposure	Outcomes measured	Principal findings summary	Ref.
1	Randomized Controlled	Ventura et al.	2012	Infants	Variable	Oral (formula)	Various experimental formulas	Acute (single testing session)	Formula type testing	Feeding behavior	Umami taste acceptance improves formula palatability	6
2		Mennella et al.	2016	Infants	50	Oral (formula)	Ad libitum (hydrolysed protein)	7 months	Extensive protein hydrolysate formula	Cognitive, motor development, feeding	Higher cognitive and motor scores; improved satiety indicators	20
3	Observational	Steiner et al. (Experimental/book chapter)	1987	Healthy term infants	40	Oral (swab/formula)	0.05 M Glutamate	Acute (initial response)	Glutamate-enriched infant formula	Study describes the innate gusto-facial reflexes to umami stimuli, establishing the physiological basis for early-life glutamate recognition.	Increased acceptance; improved motor development at 6 months	21
4		Agostoni et al.	2000	Lactating mothers	N/A	Maternal diet (natural)	Baseline dietary intake	Longitudinal (lactation)	Breast milk amino acid profiling	Free glutamate concentration in milk	Increasing free glutamate over lactation period	4
5		Larnkjær et al.,	2016	Healthy lactating mothers and their term infants	50 mother–infant dyads	Maternal diet (natural)	Baseline dietary intake	4 months (lactation)	Natural variation in free amino acids in human milk	Milk free amino acid profile; infant growth	Free glutamate was the most abundant free amino acid in human milk throughout lactation and was not associated with maternal anthropometry or adverse infant growth, supporting its physiological role in infancy.	22
6		Stegink et al.,	1972	Healthy lactating women	6	Oral (bolus)	100 mg/kg body weight	Acute (single dose)	Single oral dose of monosodium glutamate (100 mg/kg body weight)	Plasma amino acid concentrations; free amino acid levels in breast milk	Oral MSG ingestion caused a transient increase in maternal plasma glutamate, peaking within 1 h and returning to baseline by 2–3 h. No significant increase in free glutamate concentration in breast milk was observed, indicating effective maternal and mammary regulation of glutamate transfer and minimal dietary MSG transmission to the nursing infant.	29
7		Rassin et al.,	1978	Human lactating women and multiple mammalian species (comparative milk analysis: human, cow, goat, sheep, dog, cat, rat, etc.)	NA	Observational (natural)	Baseline (endogenous levels)	Longitudinal (lactation)	Observational biochemical analysis of naturally occurring milk	Concentrations of taurine and other free amino acids (including glutamate, glutamine, glycine, alanine, etc.) measured using chromatographic techniques	Human milk contained substantially higher concentrations of free taurine and free glutamate compared with most other mammalian milks. Free amino acids represented a significant proportion of total amino acids in human milk. Authors suggested that the high free amino acid content in human milk likely reflects physiological and developmental importance for the human infant, particularly for neural and metabolic development.	27
8		Zhang et al.	2013	Global milk samples	N/A	Literature Review	Narrative synthesis of levels	N/A (review)	Literature review	Free amino acid profiles	Glutamate is most abundant free amino acid across lactation	30
9		Mennella et al.	2023	Formula-fed infants	Multi-site	Oral (formula)	Various infant formula types	Longitudinal/chronic	Various infant formula types	Dietary glutamate intake, safety	High intake safe with normal growth; challenges ADI relevance	31
10		Schwartz et al.	2013	Infants	40	Oral (taste testing)	Ad libitum (taste preference)	Acute (single SESSION)	Infant taste preference testing	Taste acceptance	Preference for umami taste; linked to breastfeeding duration	23
11		Tung and Tung	1980	Term and preterm infants	Variable	Oral	60 mg/kg	Acute (single dose)	Oral MSG administration	Plasma glutamate levels	Doubling of plasma glutamate; no toxicity	28
12		Somaraki et al	2023	Term or moderate-late preterm infants (≥33 weeks gestation) enrolled in the nationwide French birth cohort ELFE (Étude Longitudinale Française depuis l’Enfance), born in 2011 across 349 maternity wards in metropolitan France.	6,979 formula-fed infants (i.e., infants receiving infant formula at about 2 months) with complete data.	Oral (Formula)	Ad libitum formula types	Chronic (up to 3.5 years)	Exposure: type of infant formula at ~2 months classified by protein hydrolysis degree: non-hydrolysed proteins (nHF), partially hydrolysed proteins (pHF, with/without “HA” label), or extensively hydrolysed proteins/amino-acid mixture (eHF/AA)	Child neurodevelopment outcomes assessed at three time-points: at ~1 year and ~3.5 years via the Child Development Inventory (CDI), at ~2 years via the MacArthur-Bates Communicative Development Inventories (MB-2), and at 3.5 years via the Picture Similarities sub-scale of the British Ability Scales (PS-3.5).	The degree of protein hydrolysis in the formula consumed at 2 months was not associated with overall neurodevelopmental scores up to 3.5 years in adjusted analyses. Some associations were observed in specific sub-domains (e.g., gross motor skills) for partially hydrolysed formula, but these were inconsistent (they did not persist across ages or sensitivity analyses). The findings support the safety of hydrolysed protein formulas in terms of overall neurodevelopment in this population.	33
13		Riedijk et al.	2007	Preterm infants	Variable	Enteral (tracer)	Stable isotope tracer	Short-term (metabolic study)	Stable isotope tracer glutamate metabolism	Oxidation rates	High first-pass metabolism; supports gut metabolism	24
14		Baker et al.,	1979	Healthy lactating women	~20–30 mothers (varied across analyses; exact *n* differed by lactational stage)	Maternal Diet	Baseline maternal diet	Longitudinal (across lactation)	Lactational stage, maternal diet, time of sampling	Free and protein-bound dicarboxylic amino acids (glutamate, glutamine) in human milk	Glutamate was the predominant dicarboxylic amino acid in human milk, with concentrations influenced primarily by stage of lactation rather than maternal dietary intake. Total glutamate content (free + bound) remained relatively stable across maternal dietary patterns, supporting endogenous regulation of milk glutamate composition.	32
15		Noorlander et al.,	2004	Human placental tissue	Not specified (laboratory histology/expression study)	Ex vivo/laboratory	N/A (immunohistochemical)	N/A (tissue study)	Immunohistochemical and molecular assessment of EAAT glutamate transporter expression in placenta	EAAT expression profiles in placental syncytiotrophoblast and fetal endothelium	Multiple EAAT transporter subtypes are expressed in human placenta; these contribute to glutamate regulatory barrier and modulate fetal amino acid supply; supports active role in limiting fetal glutamate exposure.	34
16		McIntyre et al.,	2020	Human placenta in fetal growth restriction vs. controls	25 placental samples (details in text)	Ex vivo/laboratory	N/A (gene expression/uptake)	N/A (placental study)	Examination of transporter gene expression and amino acid uptake rates by mass spectrometry and transcript analysis	Amino acid transport and uptake rates; transporter protein expression	Placentae from FGR cases show upregulated amino acid transporter genes but lower actual rates of glutamine, glutamate uptake; highlights regulatory and functional deficits relevant for fetal nutrition.	35
17		Haÿs et al.,	2007	Preterm infants receiving enteral feed	11 preterm infants	Enteral (nasogastric)	Stable isotope tracer	Acute (metabolic assessment)	Enteral stable isotope-labeled glutamate administration	Splanchnic and systemic glutamate uptake and oxidation rates; arterial–venous sampling	Up to 74% of dietary glutamate is metabolized on first pass, predominantly via oxidation; confirms gut’s major role in glutamate metabolism in preterm infants, minimizing peripheral exposure.	25
18		Battaglia et al.,	2000	Human placental and fetal liver samples	Not specified (ex vivo samples, ~12 human placentas)	Ex vivo/tracer	Tracer quantities (glutamine)	Acute (metabolic study)	Amino acid concentration profiling; tracer methodologies for glutamine and glutamate	Placental and fetal uptake, conversion rates (glutamine→glutamate); hepatic output	Fetus receives high glutamine, converted to glutamate in liver, mostly extracted by placenta and oxidized; placenta metabolizes majority of fetal plasma glutamate during late gestation and parturition.	26
19	Animal/Preclinical	Olney et al.	1969	Rodent models	Variable	Parenteral (SC/IP)	500–4,000 mg/kg	(Supra-physiologic) acute/short-term	High-dose MSG exposure	Neurotoxicity, motor impairments	Toxicity only at supra-physiologic doses; not dietarily relevant	36
20		Stegink et al.	1975	Rodent models	Variable	Oral (gavage)	1,000–4,000 mg/kg	Acute/short-term	Oral MSG administration	Plasma glutamate, toxicology	Efficient metabolism; no systemic accumulation or neurotoxicity	37
21		Food and Drug Research Laboratories, Inc. (for U.S. FDA)	1974	Pregnant mice, rats, and rabbits	NA	Oral (Gavage)	Various (FDA 71–69)	During organogenesis	Oral monosodium glutamate (FDA 71–69) administered during organogenesis	Maternal toxicity; fetal viability; external, visceral, and skeletal malformations	Oral MSG exposure did not produce teratogenic effects in mice, rats, or rabbits at the doses tested. No treatment-related increases in fetal malformations or adverse developmental outcomes were observed, supporting the absence of teratogenic risk from oral MSG exposure under experimental conditions.	39
22		Newman et al.	1973	Pregnant rhesus monkeys	N/A	Oral (dietary/gavage)	4,000 mg/kg	Late gestation	Oral MSG exposure in late gestation	Neonate brain histopathology	No neurotoxicity detected despite high dose exposure	44
23		Semprini et al.	1974	Rodent models	N/A	Oral (dietary)	High-dose dietary inclusion	Short-term	Oral MSG exposure	Neurotoxicity in the hypothalamus	Oral dosing safe	40
24		Takasaki et al.	1979	Neonatal and juvenile rats	Multiple treatment groups (exact *n* per group varied by experiment)	Oral vs. Parenteral	High-dose comparative	Early life (neonatal/juvenile)	Monosodium L-glutamate administered parenterally (subcutaneous/intraperitoneal) or orally at varying doses during early life	Somatic growth parameters (body weight gain, organ weights); general health observations	Parenteral MSG administration resulted in suppressed somatic growth and altered body weight gain, whereas oral MSG administration produced no adverse effects on growth, even at high doses. The findings demonstrate that growth impairment is route-dependent and not observed with dietary (oral) exposure.	41
25		Anantharaman K	1979	Mice (multigeneration model) (Anantharaman, 1979)	Multiple generations; group size not specified (~30 per generation typical in design)	Oral (Dietary)	Up to 6,000 mg/kg/day	Multigenerational	Dietary MSG given to maternal mice before mating through lactation, or from gestation day 14 through lactation, max 6,000 mg/kg/day	Fertility, survival, growth, litter size, physical/neural development, histopathology	No adverse effects on fertility, litter size, development; brain histology normal; high-dose oral/in utero MSG does not produce reproductive or developmental toxicity in mice over multiple generations.	42
26		Holzman et al.,	1979	Pregnant sheep	Primary cohort: 10 pregnant ewes Amino acid measurement subgroup: 7 ewes Glutamine-glutamate A–V difference subgroup: 4 ewes	In vivo (tracer)	Stable isotope tracers	Acute (surgical model)	Fetal liver glutamate metabolism	Placental glutamate handling	Placenta metabolizes fetal glutamate; protects fetus	38
27	Regulatory bodies	JECFA	1971–2022	Comprehensive	N/A	NA (regulatory studies)			Safety assessments	ADI, toxicology, human safety	MSG safe at dietary levels with ADI “not specified” status	6–9, 13, 41, 43, 44
28		JECFA	2006	Reviews	N/A				Review of glutamate safety	Metabolic compatibility	Reaffirmed low toxicity and safety	
29		JECFA	2022	Updated evaluations	N/A				Global safety data review	Toxicology, genotoxicity	Safety of flavoring agents including MSG upheld	
30		EFSA	2017	Risk assessment	N/A				Systematic review	Toxicology, neurodevelopment	Affirmed safety of glutamate at current exposure levels	
31		FDA	1995	US population	N/A				Dietary exposure assessment	Food additive safety	No identified safety concerns at permitted usage levels	
32		FSSAI	2020	India-specific	N/A				Infant food labeling precaution	Nutritional safety	Precautionary labeling; no direct toxicological safety concern	

The table summarizes key studies related to monosodium glutamate (MSG) and glutamate in infant nutrition and related models. It provides details on study types, authorship, years, populations or models examined, sample sizes, interventions or exposures, outcomes measured, principal findings, and references. This comprehensive overview includes randomized controlled trials, observational studies, animal/preclinical models, and evaluations by regulatory bodies, highlighting safety, metabolic fate, neurodevelopmental effects, and acceptance of glutamate and MSG in infant feeding contexts.

### 3.2 Randomized controlled trials

In a longitudinal RCT, infants were enrolled at birth (~0.5 months of age). The research team randomly assigned infants to receive either an extensively hydrolyzed formula (naturally higher in free glutamate due to protein hydrolysis) or a standard cow’s milk formula for 8 months (20). Cognitive and motor outcomes were assessed using standardized developmental screening tools at 8.5 months of age. Infants in the high-glutamate group demonstrated significantly improved motor and mental performance. Experimental studies in infants demonstrate that free glutamate, a naturally abundant component of human milk, is readily detected and accepted, suggesting a role in early taste learning and feeding behavior.

In a counterbalanced, within-subject, crossover RCT, 30 infants younger than 4 months were studied across three test days (6). During each session, infants were fed one of three isocaloric formulas: (i) cow’s milk formula (CMF), (ii) extensive protein hydrolysate formula (ePHF, high in free glutamate), or (iii) CMF with added free glutamate (CMF + glu) to match ePHF levels. Outcomes included intake during the first meal, satiety (measured by the intermeal interval divided by amount consumed), and behavioral signs of satiation and satiety. Infants consumed significantly less ePHF and CMF + glu compared to CMF during the first meal (*p* < 0.04 and *p* < 0.02, respectively). Both ePHF and CMF + glu led to higher satiety ratios, indicating greater postprandial satiety. No differences in later meal intakes or compensatory feeding were observed. The study concluded that free glutamate concentration in formula acutely enhances satiation and inter-meal satiety in young infants, independent of flavor changes or parental feeding behavior.

### 3.3 Observational studies

Early experimental studies of neonatal taste perception demonstrated that human infants exhibit characteristic positive facial expressions (gusto-facial reflex) in response to umami stimuli, including glutamate, indicating an innate acceptance of this taste. These studies focused on immediate sensory responses and did not assess feeding volume, satiety, or later developmental outcomes (21).

Recent cohort studies measuring free amino acids in human milk confirm that glutamate is among the most abundant, with consistently high concentrations throughout lactation. In a cohort of approximately 50 fully breastfeeding mothers, free glutamate constituted a significant component of the milk free amino acid pool, and levels showed no adverse associations with infant growth parameters (22). Intake modelling based on pooled human milk compositional data indicates that breast-fed infants receive approximately 35–40 mg/kg/day of free glutamate, a level considered physiologically normal and without evidence of clinical harm (3). In another cohort study comprising six-month-old infants (*n* = 122), taste preferences were evaluated using standardized aqueous taste solutions (sweet, salty, bitter, and umami/MSG) in a double-blinded setup (23). The primary outcomes were duration and frequency of acceptance responses. At 6 months, infants preferred sweet, salty, and umami solutions over water and were indifferent to sour and bitter solutions. Notably, a positive correlation (*r* = 0.42) was observed between breastfeeding duration and umami preference, suggesting an early flavor-programming effect driven by glutamate in breast milk.

A metabolic study in preterm infants, employing stable-isotope-labeled glutamate tracers delivered enterally to quantify glutamate metabolism directly, was undertaken (24). Blood samples were obtained via heel stick to represent the systemic circulation at multiple time points. The results indicated that the splanchnic bed (gut and liver) metabolized approximately 74% of dietary glutamate during first-pass, with the majority being oxidized to CO₂. Minimal labeled glutamate reached peripheral circulation, reinforcing the concept that dietary glutamate’s principal role in neonates is as an intestinal energy substrate, not as a systemic amino acid pool contributor.

Similarly, in 2007, researchers found that premature infants possess a highly efficient mechanism for processing dietary glutamate, characterized by extensive extraction during its “first pass” through the gut and liver (the splanchnic bed). This high rate of extraction ensures that the vast majority of dietary glutamate is metabolized, primarily oxidized, within these organs, thereby preventing a significant systemic increase in blood-free glutamate. This finding is critical because it demonstrates that the splanchnic bed acts as a robust metabolic barrier, protecting developing neural and systemic tissues from potential fluctuations in plasma glutamate levels following oral intake (25). The findings highlight glutamate’s predominant role in gut energy metabolism and its limited contribution to peripheral amino acid pools, reinforcing its safety and metabolic importance in enteral infant formulas.

Investigating sheep placental and fetal liver samples, Battaglia et al. used amino acid concentration profiling and tracer methodologies to elucidate glutamine and glutamate exchange during late gestation (26). Their data showed that the fetus receives high levels of glutamine, which is converted in the liver to glutamate and then primarily extracted by the placenta for oxidation. During parturition, shifts in hepatic output and placental uptake reflected hormonal transitions, with placental glutamate metabolism accounting for the majority of fetal plasma glutamate clearance *in utero*. Rassin et al. compared milk from different species and found that human milk contains 6–10 times more free glutamate than cow’s milk or milk from other mammals (27). Importantly, this per-body-weight intake amount is higher than that of adults, highlighting that high glutamate exposure is a physiological requirement during infancy.

Pioneering comparative work by Tung and Tung conducted in 1980, measured serum free amino acid concentrations after oral glutamate intake in healthy infants, preterm neonates, and adults (28). Subjects ingested an oral dose of MSG equivalent to 150 mg/kg bw, administered within a typical feeding matrix (infant formula for infants, rice porridge for adults). Serum samples taken at intervals post-ingestion revealed a transient 2-fold increase in serum glutamate in infants, peaking approximately 30 min after intake and returning to baseline within 60 min. Adults showed a lower and shorter increase in duration (~1.3-fold). No adverse clinical effects were reported in any group, including preterm infants. The differences in serum glutamate levels were attributed to variations in metabolic rates and food matrix between infants and adults. These data indicate that infants, even pre-terms, efficiently metabolize substantial oral doses of free glutamate without harmful accumulation. In a prospective cohort of healthy lactating mothers, the concentrations of free glutamine and glutamic acid were monitored in breast milk over 3 months postpartum (4). Samples were analyzed using advanced chromatography. Free glutamate steadily increased from colostrum through mature milk, independently of maternal diet or MSG intake, suggesting a tightly regulated, endogenous mechanism for glutamate provision in human milk to support neonatal development.

Pioneering study by Stegink et al. in 1972 investigated the impact of a single oral dose of MSG (6 g) on plasma and breast milk amino acid concentrations in lactating women (29). The study monitored plasma and milk levels of free glutamate, glutamine, and alanine before and after MSG ingestion. Results showed that maternal plasma glutamate levels increased significantly following MSG intake, peaking approximately twice baseline levels within 2 h and returning to baseline by 4 h. Despite this transient elevation in plasma, the concentrations of glutamate, glutamine, and alanine in breast milk remained unchanged over the monitoring period. This suggests that maternal ingestion of pharmacological doses of MSG does not significantly alter the amino acid composition of breast milk. No participants experienced adverse effects related to the intervention.

A critical systematic review of amino acid profiles in term and preterm human milk (across multiple countries and varying lactational ages) confirmed that glutamate is the most abundant free amino acid at all stages of lactation (30). Another multi-race observational study assessed glutamic acid intake in infants fed different types of formula, with consumption logs and formula compositional analysis (31). Infants fed extensively CMF and protein hydrolyzed formulas ingested free glutamate levels several-fold higher than the current EFSA ADI of 30 mg/kg bw/day. The ADI applies to the general population, except for infants under 16 weeks in the EU. Growth monitoring revealed no safety concerns, and the authors questioned the appropriateness of ADIs for infants, advocating for the development of infant-specific intake guidelines. This high level is consistent throughout the lactation period. Foundational works by Baker et al. (1979) reported that the total glutamate content, both free and bound, in human milk is approximately 230.0 mg/dL. For a breastfed infant, this translates to an average free glutamate intake of roughly 36 mg/kg bw/day, which is equivalent to 46 mg/kg bw/day of MSG. In comparison, total daily protein-bound glutamate intake is approximately 357 mg/kg bw/day (32).

In a French nationwide ELFE birth cohort, neurodevelopmental outcomes in cognitive, motor, language, social, and self-help domains were examined between 1 and 3.5 years in groups fed non-hydrolyzed, partially hydrolyzed, or extensively hydrolyzed (high-glutamate) formulas (33). No significant differences in global neurodevelopmental scores were found between formula types post-adjustment for confounders, with only minor, inconsistent domain differences in social and fine motor outcomes that were not robust in sensitivity analyses.

Human placental tissue was analyzed for expression of EAAT (Excitatory Amino Acid Transporter) subtypes using immunohistochemistry and transcript profiling (34). The study mapped the localization of EAAT1 and EAAT2 in syncytiotrophoblasts and EAAT3 in fetal endothelium, implicating these transporters in the regulation and maintenance of the maternal-fetal glutamate exchange barrier function. The results support an active role for the placenta in modulating fetal amino acid supply and in protecting the fetus from glutamate overload. Using mass spectrometry of placental tissue from cases of fetal growth restriction (FGR) and controls, this study quantified amino acid uptake rates and transporter protein expression (35). Although FGR placentae exhibited upregulated transporter genes (LAT1, LAT2, SNAT5 for glutamine; EAAT1 for glutamate), actual rates of glutamine and glutamate uptake were reduced per milligram of protein. The functional deficit in the presence of increased transporter activity highlights potential therapeutic targets and supports the clinical relevance of glutamate metabolism for fetal development.

### 3.4 Preclinical animal studies

Olney’s seminal rodent studies established that neonatal mice exposed to high parenteral doses of MSG (0.5 to 4 mg/g) exhibited vacuolar degeneration and neuronal loss within the arcuate nucleus and hypothalamus, effects associated with neuroexcitotoxicity (36). This neuronal damage was dose-dependent and rapid, and was interpreted as evidence that high levels of circulating glutamate (from injections) were neurotoxic to immature brain tissue. Following this, another foundational study by Stegink et al. (1975) reported that the placental transfer of glutamate and its metabolites in pregnant rhesus monkeys to assess fetal exposure to maternal glutamate (37). Using radioactive tracer infusion of glutamate, the study found that 69–88% of the radiolabel in maternal plasma remained associated with glutamate, while 10–22% was metabolized to glucose. In fetal plasma, over 80% of the radiolabel was detected as glucose and lactate, with less than 2% as glutamate, indicating minimal direct glutamate transfer. Even when maternal plasma glutamate was elevated 10 to 20-fold (60 to 100 μmol/100 mL), fetal glutamate levels remained unchanged. Only at extremely high maternal glutamate concentrations (about 70 times normal) was transfer to the fetal circulation detected. Furthermore, fetal glutamate infusion at doses up to 5 g/kg bw increased fetal plasma glutamate levels but resulted in minimal transfer to the maternal circulation. The metabolites glucose and lactate crossed the placenta readily in both directions, highlighting placental metabolism as a barrier to direct fetal exposure to glutamate.

In a pioneering study conducted in 1979, the amino acid uptake by the uterus and the placental glutamine-glutamate balance were reported in pregnant ewes, aiming to elucidate fetal nutrient supply and placental metabolic roles in ruminant pregnancy (38). Using *in vivo* sampling and arteriovenous difference measurements, the study quantified uterine amino acid uptake during mid- to late gestation. The uterine uptake of glutamine significantly exceeded that of glutamate, revealing a net release of glutamate by the placenta. These findings indicated active placental metabolism converting maternal glutamine to glutamate, which then crossed the placental barrier to supply the fetus. The glutamine-glutamate shuttle between the placenta and fetus was thus identified as a crucial amino acid exchange mechanism essential for fetal nitrogen balance and growth.

Food and Drug Research Laboratories (1974a) exposed pregnant mice to dietary MSG at doses of 0, 11.4, 53.0, 246, or 1,140 mg/kg bw for 10 days during gestation. No adverse effects were observed on implantation, maternal survival, fetal survival, resorptions, fetal weight, litter size, or skeletal/soft-tissue development (39). Researchers fed mice and rats diets containing 0, 1, or 2% MSG, with or without added vitamins, over two generations. No histopathological abnormalities, including in brain tissue, were found (40). Another pioneer comprehensive evaluation in 1979, reported the effects of both parenteral and oral MSG administration on somatic growth in rats. The researchers observed that while high-dose parenteral administration had specific effects, oral intake, representative of dietary consumption, did not adversely impact normal growth patterns or developmental milestones. These findings contribute to the evidence base suggesting that the route of administration is a critical factor in determining the metabolic and physiological response to MSG (41).

In another seminal multi-generation study by Anantharaman (1979) assessed the reproductive performance and developmental effects of MSG in mice across three generations (F1 to F3). Mice were administered dietary MSG at concentrations of 1% or 4%, with intakes reaching up to 6,000 mg/kg bw/day in males and 7,200 mg/kg bw/day in females, and as high as 25 g/kg bw/day during lactation (37). Evaluations included fertility, gestation length, litter size, pup survival, and physical development milestones. Additionally, histopathological analysis of neurological tissue from the first- and third-generation offspring revealed no gross or microscopic abnormalities or reductions in neural density. Fertility, viability, and lactation indices remained within normal ranges, suggesting that high-dose in utero and dietary MSG exposure does not impair reproductive performance or cause neurodevelopmental toxicity (42).

A two-generation OECD 416-compliant rat study used feed containing 5,000, 15,000, or 50,000 mg/kg MSG (43). No observed adverse effect levels (NOAELs) were set at 939 mg/kg bw/day (males) and 1,039 mg/kg bw/day (females) by the author based on kidney weight changes without histopathological findings, while developmental NOAELs were 3,131 and 3,496 mg/kg bw/day identified by JECFA. Collectively, these reproductive and developmental studies demonstrate no evidence of adverse effects attributable to MSG exposure.

Using pregnant rhesus monkeys, researchers administered oral MSG at doses up to 4 g/kg bw daily during late gestation (44). Fetal plasma glutamate was measured, and comprehensive histopathology of neonatal brains was undertaken. Results demonstrated unchanged fetal plasma glutamate levels and absence of brain abnormalities, supporting both limited placental transfer of glutamate and effective fetal neuroprotection in primates, even at supraphysiological MSG exposures.

### 3.5 Regulatory evaluations by JECFA, EFSA, FSSAI

Rigorous multi-decade toxicology and metabolism reviews by the JECFA led to MSG and glutamate being transitioned from a specified ADI to “ADI not specified,” denoting extremely low toxicity (5, 7, 17). Recent JECFA (2022) reviews, incorporating human and animal data, exposure studies, and metabolism, have upheld the safe use profile for all populations, including infants and pregnant women (43). In 2017, EFSA performed parallel evaluations, confirming safety and additionally endorsing new manufacturing methods for glutamate salts (5). Meanwhile, FSSAI in India implemented precautionary labeling for infants on all food products containing added MSG as a population-specific safeguard (15).

In 2023, a Chinese study by Zhou et al. reported an evaluation of the use, safety, and dietary exposure levels of glutamates, including MSG, in the food supplies (45). The report notes that glutamate and MSG are widely used as flavor enhancers in various food products, including processed meats, sauces, soups, snacks, and hydrolyzed vegetable proteins. Analytical measurements across various food sources, including mixed meals, powdered formulas, and dairy products, indicate average glutamate levels ranging from approximately 2.29 mg/kg in milk/dairy products to 5.12 mg/kg in mixed meals, with powdered infant formulas averaging 3.89 mg/kg. The overall mean daily dietary exposure in children aged 2–5 years was well below the Acceptable Daily Intake (ADI) of 120 mg/kg bw (expressed as glutamic acid), as established by JECFA in 1974 (46). The highest contributors to dietary glutamate exposure were mixed meals, followed by milk and dairy, and powdered formula.

## 4 Discussion

This review evaluates the metabolic, functional, and safety dimensions of dietary glutamate in infancy by synthesizing evidence from RCTs, observational studies, animal research, metabolic tracer studies, and regulatory evaluations. The principal outcomes confirm that free glutamate is a prominent, physiologically abundant amino acid in human milk and in specific hydrolyzed infant formulas, is efficiently metabolized by the neonatal gut and liver, and has a favorable safety profile at dietary intakes relevant to infants and young children.

### 4.1 Biological and metabolic roles of glutamate in infancy

Human milk is a rich natural source of glutamate for infants. Both protein-bound and free forms of glutamate are present, but free glutamate is abundant compared to other amino acids. In 2013, Zhang et al. reviewed global data and confirmed that glutamate is the most concentrated free amino acid in human milk, irrespective of the mother’s geographical location (30). The findings of Rassin et al. highlight a unique evolutionary adaptation: human infants are naturally exposed to 6–10 times more free glutamate than other mammals (27) This high per-body-weight exposure suggests that elevated glutamate levels are not only safe but may be a fundamental physiological requirement for neonatal development and metabolic health.

The early data from Tung and Tung (1980) provide foundational human evidence that infants and preterm neonates are not uniquely susceptible to systemic glutamate accumulation compared with adults (28). While infants exhibit a more pronounced, transient rise in serum glutamate, the return to baseline within 1 hour, paired with the absence of clinical sequelae, aligns with the high splanchnic extraction rates observed in more recent tracer studies (25). When synthesized, these data suggest that the infant’s splanchnic bed acts as an effective metabolic ‘buffer.’ Unlike adults, the higher peak in infants likely reflects a transitional physiological stage of gut absorption and metabolism rather than an inability to safely manage dietary glutamate, a conclusion supported by the lack of adverse outcomes even at the high doses administered in this study (28). The absence of clinical or biochemical adverse effects supports the metabolic safety of free glutamate at dietary levels encountered in breast milk and hydrolyzed infant formulas. This early work underpins the metabolic rationale for the lack of toxicity or developmental concerns associated with dietary glutamate observed in later RCTs and cohort studies. It also corroborates subsequent findings of extensive splanchnic extraction and glutamate oxidation, thereby limiting peripheral exposure. The study’s design, involving ingestion within standard feeding substrates rather than pure bolus water doses, adds ecological validity to the metabolic safety assessment. Together with later regulatory reviews, Tung and Tung’s results reinforce the scientific basis for current safe intake levels of glutamate in infant nutrition.

According to the Federation of American Societies for Experimental Biology, infants fed formula based on casein hydrolysates (the formula marketed especially for the infants of allergic to intact proteins) could consume approximately 247 mg/kg bw/day of free glutamate. This amount is many times higher than that in breast milk, but it causes no adverse physiological effects (47).

The apparent discrepancy between Mennella et al. (2016), who reported potential cognitive benefits (20), and Somaraki et al. (2023), who found no global neurodevelopmental impact (33), may be attributed to the specificity of the assessment tools used. Mennella’s focus on the ‘gut-brain-flavor’ axis suggests that glutamate may enhance satiation and certain cognitive associations, whereas Somaraki’s broader outcomes indicate that at standard dietary levels, glutamate does not disrupt the fundamental trajectory of neurodevelopment. These findings are not necessarily contradictory but rather reflect different facets of the nutrient’s functional role. We suggest that the “cognitive benefit” observed by Mennella may be linked to specific metabolic signaling in the gut-brain axis, whereas the “no effect” found by Somaraki may reflect the resilience of global neurodevelopmental markers at standard exposure levels.

### 4.2 Glutamate, taste perception, and feeding behavior

Exclusive breastfeeding has become rarer in recent decades, particularly among urban and professional populations (48). In such cases, infant formula and dietary supplements are necessary for promoting newborn growth and development. Improving the palatability of these supplements is crucial, as early taste sensations significantly influence eating behavior and dietary acceptability. The inclusion of glutamate, a naturally occurring amino acid found in human milk, can increase the umami taste and acceptability of baby meals while maintaining safety. Behavioral taste studies have shown that infants can detect glutamate from the earliest stages of life. Using facial expression analysis, researchers observed that newborns readily accepted vegetable soup containing added glutamate, suggesting an innate preference for the umami taste (21).

Schwartz et al. (2013) reported on taste preferences in six-month-old babies. Using aqueous solutions of different tastes, they found that infants preferred sweet, salty, and umami (MSG) solutions over water. Interestingly, the duration of breastfeeding correlated positively with acceptance of umami, suggesting that the naturally higher glutamate content of breast milk compared to formula milk may play a role in early flavour programming (23).

Studies on feeding behavior also suggest a potential role for glutamate in regulating appetite. In 2012, a team of scientists examined three formula types: Cow’s Milk Formula (CMF), an Extensive Protein Hydrolysate Formula (ePHF) that was naturally high in free glutamate (~1,099 mg/L), and CMF + MSG, which was fortified to match the free glutamate content of ePHF. Infants were fed each formula type in a controlled crossover design. The observation was that intake volume was significantly lower with CMF + MSG and ePHF compared to CMF alone. The time to the next feed per unit of formula consumed was considerably prolonged for CMF + MSG and ePHF (6). Later in 2016, Mennella et al. extended these findings to developmental outcomes. In a longitudinal study, infants fed ePHF achieved higher motor and cognitive scores than CMF-fed infants, suggesting that greater early glutamate exposure may contribute to better developmental milestones (20).

### 4.3 Neurodevelopment and cognitive outcomes

Direct clinical trials in pregnant women or infants to assess the reproductive and developmental safety of food additives such as MSG are not feasible. For this reason, preclinical studies conducted on animals are accepted in accordance with the defined protocol. Reproductive and developmental toxicology studies in rodents remain the internationally recognized gold standard. When considered collectively, early controlled animal studies provide essential context for interpreting developmental exposure to dietary glutamate (39–41). Across gestational and multigenerational murine models, dietary MSG, even at concentrations far exceeding typical human intake, did not adversely affect fetal growth, reproductive performance, or major organ histology, including the brain. These convergent findings support the concept that orally ingested glutamate is efficiently metabolised and tightly regulated, limiting systemic and neurodevelopmental exposure, and are consistent with human and regulatory evidence indicating the developmental safety of dietary glutamate and glutamine (Table 1).

The foundational multigenerational reproductive toxicity study by Anantharaman provides robust evidence that sustained dietary MSG exposure in mice is without adverse neurodevelopmental or reproductive effects (42). Unlike acute parenteral administration models that induce hypothalamic neuronal lesions, this chronic dietary model mimics typical oral exposure and reflects more physiologically relevant conditions. The study demonstrated consistent growth profiles, reproductive outcomes, and brain histology across F1-F3 generations at doses up to 6,000 mg/kg bw/day in males and 7,200 mg/kg bw/day in females. This research challenges concerns extrapolated from high-dose injection studies and highlights the importance of exposure route, dose, and duration in toxicological assessments of glutamate. The maintained neuronal density and lack of neurodegeneration in the arcuate and other hypothalamic nuclei further reassure that dietary glutamate does not cause the neuropathological damage observed in neonatal MSG-injection models. Collectively, the Anantharaman study strengthens the preclinical evidence base supporting the metabolic compatibility and safety of MSG at dietary levels, underpinning current regulatory positions and clinical understanding of glutamate’s role in infant nutrition (42).

### 4.4 Blood–brain barrier (BBB) function in infancy

The human brain is protected very early in life by specialized barrier systems, most importantly the BBB. These barriers form during embryogenesis and are functionally active long before birth. Experimental and clinical evidence show that the BBB is functionally competent at birth and plays a vital role in protecting the developing brain. Saunders and colleagues have emphasized that barrier mechanisms in the fetal brain are established during early development, preventing potentially harmful substances in the maternal circulation from entering the fetal CNS (49). Similarly, in 2017, it was reported that the BBB is already operational by the end of the first trimester, complementing placental protection by excluding xenobiotics and tightly regulating the brain’s internal environment (50).

Detailed mapping of fetal vascular development in mice illustrates the stepwise acquisition of barrier features, stating that endothelial precursor cells form a vascular network by gestational day 10, pericytes stabilize these vessels by day 12, and by day 18, tight junction proteins such as claudin-5 are expressed, reflecting maturation of barrier function (51). Daneman et al. emphasized through their experiments that pericytes, not astrocytes, are the key inducers of BBB integrity during embryogenesis, underscoring that barrier formation is a prenatal event rather than a postnatal phenomenon (52).

Importantly, studies in both humans and animal models converge on the conclusion that at birth, the BBB is structurally and functionally effective in regulating permeability. Ek and colleagues provided comprehensive evidence that barrier mechanisms, including the BBB and blood-cerebrospinal fluid barrier, are present and active during gestation, ensuring selective permeability and contributing to neuroprotection against toxins (53). Their work emphasized that the developing brain is not freely accessible to circulating neurotoxicants, but instead is protected by functional barriers that mature progressively *in utero*.

This developmental perspective is directly relevant to the metabolism and handling of dietary glutamate. Reviews by international bodies, including the FAO/WHO JECFA, have consistently concluded that oral intake of MSG is safe, with no evidence of increased brain glutamate levels in neonates or transfer through maternal milk at dietary exposures (54).

The placenta and BBBs effectively limit fetal exposure, and the placenta’s efficient uptake and metabolism of glutamate further minimize risks. Thus, the scientific consensus is that the neonatal brain is well protected against fluctuations in systemic glutamate, even during sensitive developmental windows. Endothelial cells forming the BBB possess Na-dependent transport systems on the abluminal side to remove glutamate from the extracellular fluid of the brain (55, 56). Removing glutamate from the synaptic cleft by EAAT1-4 is essential for preventing brain excitotoxicity. It has been reported from human-based mapping of EAAT1–EAAT4 during brain development that each EAAT subtype has a distinct developmental course. Among these, the EAAT2 is a prominent one, facilitating efficient glutamate turnover across brain regions from early gestation. Later, a temporal shift in the localizations of EAAT1 and EAAT3 hints at roles in developmental regulation and neuronal maturation. These findings lay the groundwork for understanding how glutamate transport dynamics influence neurodevelopment and how disruptions to these dynamics might contribute to neurodevelopmental conditions (57). Glutamate is either metabolized within endothelial cells or transported into the blood via luminal facilitative carriers. Plasma glutamate levels required to cause neurotoxicity in rodents (100–300 μmol/dL in neonates) are far above those observed in humans, even after large oral MSG doses of 150 mg/kg bw (9, 17). Moreover, previous studies have shown no correlation between plasma and milk glutamate concentrations in lactating women (3). Mechanistically, free glutamate in milk is mainly synthesized within mammary epithelial cells rather than transferred directly from plasma. Mammary glands actively uptake leucine via the LAT1 transporter, which is subsequently transaminated to produce glutamate, leading to consistently high and stable milk glutamate levels (58). This tightly controlled regulation is conserved across populations and is thought to reflect the physiological requirement of the breastfed infant, whose splanchnic tissues efficiently utilize dietary glutamate without elevating systemic plasma levels.

Fetal hypoxic–ischemic asphyxia can lead to a vulnerable situation where the brain becomes prone to glutamate neurotoxicity. Although the findings are from preclinical models, the enzyme Glutamate-oxaloacetate transaminase (GOT) metabolizes glutamate into *α*-ketoglutarate and oxaloacetate. Pérez-Mato et al. investigated whether endogenously produced GOT in fetal blood can serve as a natural neuroprotective buffer against glutamate overload (59). Thus, when exposed to asphyxiating conditions around birth, healthy fetuses appear to mount a protective GOT response, converting glutamate to α-ketoglutarate and oxaloacetate. Overall, the available evidence demonstrates that the BBB effectively restricts the passage of glutamate from entering into the brain, maintaining the stability of the neural microenvironment during development.

### 4.5 Placental handling, Fetal exposure, and blood–brain barrier

Pregnancy requires increases in various amino acid concentrations to meet the needs of both placental and fetal tissues, especially during late gestation (60). Animal studies have consistently demonstrated that the placenta functions as an efficient barrier to the transfer of glutamate from mother to fetus. The placenta not only transports amino acids from mother to fetus but also metabolizes them by interconverting non-essential and essential amino acids to maintain net flux in line with fetal demands (38, 61). The enzymatic activities and transporter expression in the placenta support this exchange. There is a notable conversion of glutamate to glutamine (through glutamine synthetase) or vice versa, depending on fetal demands, thus maintaining homeostasis in both compartments (35). After the placental uptake, glutamine is transferred through the umbilical vein to the fetal circulation (62). The fetal liver and other tissues then metabolize glutamine into glutamate (via glutaminase), supporting nitrogen balance and energy requirements during fetal development.

As Stegink et al. (1972) observed that breast milk glutamate levels remain stable despite maternal hyperglutamatermia, which aligns with broader physiological evidence indicating that the placenta is an effective metabolic barrier. Much like the placenta prevents significant fetal glutamate exposure by converting maternal glutamate into glutamine through active enzymatic processes, the mammary gland appears to function as a homeostatic regulator (29, 37). When synthesized with the high first-pass extraction rates observed in the neonatal splanchnic bed (24, 25), these findings collectively demonstrate that developing tissues are protected from systemic glutamate fluctuations through multi-level metabolic buffering. This mechanistic evidence clarifies why maternal dietary MSG intake does not translate to neonatal excitotoxic risk, providing a robust physiological basis for the ‘ADI not specified’ regulatory status.

Holzman et al.’s findings provide critical experimental evidence for the concept that the placenta regulates fetal amino acid delivery via glutamine-glutamate cycling (38). The greater uterine glutamine uptake, coupled with placental glutamate release, supports a model in which the placenta functions not merely as a passive conduit but also actively metabolizes amino acids to tailor fetal nutrient exposure. This metabolic interplay likely contributes to fetal growth and developmental programming. Although initially studied in sheep, these principles are highly relevant to human placental physiology, as subsequent studies have identified similar glutamine-glutamate shuttle mechanisms in the human placenta. This study reinforces the importance of placental amino acid transport and metabolism in fetal nutrition and supports clinical interest in glutamate’s role in perinatal development.

The early evidence by Baker et al. (1979) that breast milk glutamate concentrations remain unaffected despite marked fluctuations in maternal plasma following MSG intake suggests the mammary gland acts as a homeostatic ‘buffer.’ This regulatory mechanism is critical, as it ensures that the neonate’s primary nutritional source remains unaffected by maternal dietary choices. When analyzed alongside the high splanchnic extraction rates identified in neonatal tracer studies (24, 25), this stable milk profile reinforces the conclusion that the neonatal system is structurally protected from systemic glutamate loading, supporting the ‘ADI not specified’ safety classification (32). These studies address concerns regarding the potential transfer of dietary glutamate to infants via breast milk and support the metabolic safety of MSG consumption during lactation. The lack of changes in breast milk glutamate concentrations aligns with other metabolic studies, indicating efficient maternal tissue metabolism and placental barriers that limit fetal and neonatal exposure to fluctuating plasma glutamate levels.

Rodent study assessing the effect of oral MSG dosing on hypothalamic morphology. Animals received diet-relevant oral doses of MSG throughout gestation and postnatally (40). Semprini et al. found no lesions in the arcuate nucleus even at a high dose (2%) of MSG in the diet. They stated that only parenteral, extremely high doses produced lesions in the arcuate nucleus; physiologically realistic oral doses did not cause structural neurotoxicity found in Olney’s studies (36). These findings reinforce the importance of exposure route and dose in risk assessment, and support the safe profile of MSG at typical dietary intakes (36, 40).

By demonstrating that even high oral MSG doses do not affect breast milk composition, the study contributes to the evidence base, reassuring breastfeeding mothers and their infants about the safety of MSG intake. This is particularly relevant when considering free glutamate in the diet as a standard component of human milk and infant feeding.

Examination of human placental tissue from normal and intrauterine growth-restricted (IUGR) pregnancies revealed discrete localization patterns of EAAT transporters. These patterns suggest that EAAT1 and EAAT2 regulate glutamate exchange at the maternal-fetal interface, while EAAT3 supports fetal vascular transport, with broader trophoblast expression early in gestation (34). No differences in distribution were observed between normal and IUGR placentae, suggesting that any glutamate transport alterations in IUGR are likely functional rather than structural. In a recent review, Wu et al. (2015) comprehensively described the efficient transport and interconversion of glutamate and glutamine between the maternal placenta and the fetus during late gestation (62).

Scientific discussions on MSG safety frequently address the BBB’s developmental state in early life. Recent evidence indicates that barrier properties are established during embryogenesis and function well before birth. Together, these findings underscore the importance of considering developmental physiology when assessing potential neurodevelopmental risks, despite current data indicating that the infant brain is effectively protected under normal dietary exposures (63). The placenta and BBB work to protect the fetus from potentially toxic substances, which can have long-term pathological consequences. To date, our understanding of the development and function of this barrier has been limited, leading many to believe that the fetal BBB is immature, if not altogether absent. By birth, humans already have a structurally and functionally effective BBB that prevents most harmful substances from entering the brain. Prenatal maturation, driven by the formation of endothelial tight junctions and the stabilization of pericytes, ensures that the newborn brain is protected from systemic fluctuations. However, the barrier continues to refine its transport and regulatory roles after birth.

MSG is structurally and functionally identical to naturally occurring L-glutamate found in breast milk and dietary proteins. The body does not distinguish between the two sources in terms of absorption, metabolism, or physiological role. However, the key difference lies in the exposure rate and concentration. Free glutamate from MSG is absorbed more rapidly than protein-bound glutamate, which must first be hydrolyzed (54, 64). However, typical dietary doses do not appear to elevate systemic levels beyond normal physiological ranges.

### 4.6 Regulatory evaluations and safety consensus

In 1971, JECFA conducted its first comprehensive safety evaluation of MSG, reviewing over 100 scientific studies that were then available. The focus at the time was primarily on high-dose pharmacological effects in animal models, which were helpful in hazard identification but poorly representative of normal human dietary exposures. Despite observing some transient effects in experimental systems, the committee concluded there was insufficient evidence of long-term toxicity in humans. Infants under 1 year of age were excluded from the ADI of 0–120 mg/kg bw for MSG. Three years later, JECFA re-examined its MSG evaluation, analyzing 144 more studies. At the second JECFA evaluation, ADI 120 mg/kg bw was allocated as glutamic acid. This corresponds to 152 mg/kg bw as MSG. So, actually, the ADI was relaxed. Still, the committee made a significant improvement by declaring that the ADI was inappropriate for infants younger than 12 weeks, following JECFA’s general principle stating that “It is prudent that foods intended for infants under 12 weeks should contain no additives at all.”

A significant shift in JECFA’s regulatory strategy occurred later in 1987. The committee concluded that MSG did not pose a significant health risk when used at levels required for its intended scientific function, following a thorough review of 237 studies. The choice was supported by human pharmacokinetic data showing that the food matrix influenced peak plasma glutamate levels more than the amount of MSG consumed. Furthermore, according to their report, infants and adults metabolize MSG similarly (17). As a result, MSG and its related salts were reclassified by JECFA under the heading of “ADI not specified,” which is reserved for food additives with extremely low toxicity and for which a numerical ADI is not necessary. This designation considered MSG’s safety profile, as well as the fact that it naturally occurs in many high-protein foods and contributes to the total dietary intake of glutamate. Based on the new knowledge of neonatal metabolic capacity, the prior restriction on use in infants younger than 12 weeks was also lifted. JECFA, however, reaffirmed its general warning that food additives used in infant foods should be used sparingly (7).

In 2006, JECFA conducted another toxicological and metabolic review of glutamic acid and its salts, reinforcing its 1987 findings. The committee observed that L-glutamic acid is a naturally occurring component of dietary proteins, as are its monosodium, calcium, magnesium, potassium, and ammonium salts. As a result, their use as flavoring agents was considered nutritionally and metabolically compatible with a typical amino acid intake (65). Since their use at current levels posed no health risks, JECFA retained this group’s “ADI not specified” status.

As part of a broader re-evaluation of amino acid-derived flavoring agents, JECFA reaffirmed its safety assessment of glutamic acid and its salts, including MSG, in 2022. The committee reviewed the most recent information on dietary exposure, genotoxicity, metabolism, and absorption in various populations. No new toxicological issues were found. Notably, the review upheld the “ADI not specified” designation, arguing that glutamate salts do not present health risks, even to vulnerable groups, when used as flavoring agents. Twenty-six related amino acid compounds were included in the evaluation. The results of the combined analysis supported the continued safe use of MSG in food (43).

FSANZ published a Technical Report on MSG in 2003, which reviews the safety of MSG and glutamates as food additives (9). The report concluded that there was no convincing evidence that MSG causes systemic reactions leading to serious illness or mortality. Current FSANZ guidance notes that glutamates, including those naturally occurring in foods such as breast milk, are chemically equivalent, and that glutamates are authorized as food additives under good manufacturing practice (GMP) in Australia and New Zealand. Food safety authorities, including FSANZ, JECFA, EFSA, and the US FDA, have converged on the conclusion that glutamates do not pose a safety concern when used as intended in foods.

## 5 Conclusion

Extensive evaluation by JECFA and other leading international agencies, including EFSA, FSANZ, the US FDA, and the FSSAI in India, has consistently affirmed the safety of MSG. In fact, MSG is generally approved for use under Good Manufacturing Practices (GMP) except in the EU, and expert risk assessment bodies, including JECFA, have not set specific numerical limits or ADIs except EFSA. This consensus is supported by physiological evidence. Human breast milk naturally contains high concentrations of glutamate, and infants metabolize it effectively, comparable to adults. Studies on glutamate metabolism in infants, as well as investigations into BBB function, demonstrate that systemic glutamate does not cross the brain in appreciable amounts, with the infant BBB functioning as effectively as in adults.

It is worth noting that the methodological limitations of animal studies, including the use of excessive doses, non-oral routes of administration, gavage administration without food, species-specific sensitivities, and an impermeable placenta in primates, limit their relevance to human dietary exposure. No human or primate data have replicated the adverse rodent findings. Research in pregnant primates confirms that the placenta is essentially impermeable to glutamate, providing an additional metabolic and enzymatic shelter for the fetus.

The safety profile of dietary MSG during early development is governed by three distinct physiological compartments. First, in pregnant women and the fetus, the human placenta serves as an active metabolic barrier, where high-affinity excitatory amino acid transporters (EAATs) prevent maternal-fetal flux, even under high-bolus conditions (37, 66). Second, for lactating women and neonates, the mammary gland maintains homeostatic control over free glutamate concentrations in human milk, ensuring that maternal MSG ingestion does not result in neonatal exposure spikes (3, 58). Finally, for infants receiving milk formula, while exogenous glutamate levels can be higher than those in breast milk, the rapid first-pass metabolism by the infant gut mucosa, which utilizes over 95% of ingested glutamate as a primary fuel source, effectively limits systemic bioavailability (67). By compartmentalizing the evidence across these three windows, it becomes clear that evolutionary maternal–infant barriers are specialized to maintain stable glutamate levels despite dietary fluctuations.

In India, MSG is permitted for use under GMP, and no numerical upper limit has been established for its consumption. Nevertheless, the FSSAI requires a mandatory warning label advising caution for use in infants and pregnant women. It is important to note that, in accordance with regulatory standards, the majority of permitted food additives are not permitted in infant foods, like the Codex Standard for Infant Formula and Formulas for Special Medical Purposes Intended for Infants, which permits only a limited number of specially authorised food additives (68). The specific requirement of a warning label for MSG is therefore distinctive, as other generally permitted additives that are not permitted for infant formula are not subject to similar provisions. The available scientific and regulatory evidence does not support the need for such a warning, indicating that this measure is not aligned with current risk assessment outcomes. Furthermore, glutamic acid and its salts are permitted in infant nutrition under the Foods for Special Medical Purposes (FSMP) category, as specified in the Food Safety and Standards (Foods for Infant Nutrition) Regulations, 2020 (69). In addition, it can be inferred that FSS (Food for Infant Nutrition) permits glutamic acid in infant formula because it allows amino acids ordinarily found in human milk as optional ingredients. This regulatory allowance acknowledges their potential role in meeting infants’ specific nutritional requirements. The totality of current evidence thus supports the conclusion that MSG is safe for the general population, including infants and pregnant women.

## Data Availability

The original contributions presented in the study are included in the article/supplementary material, further inquiries can be directed to the corresponding author/s.
